# Current evidence and clinical relevance of drug-microbiota interactions in inflammatory bowel disease

**DOI:** 10.3389/fmicb.2023.1107976

**Published:** 2023-02-23

**Authors:** Heike E. F. Becker, Karlijn Demers, Luc J. J. Derijks, Daisy M. A. E. Jonkers, John Penders

**Affiliations:** ^1^Division Gastroenterology-Hepatology, Department of Internal Medicine, NUTRIM School of Translational Research in Metabolism, Maastricht University Medical Centre+, Maastricht, Netherlands; ^2^Department of Medical Microbiology, Infectious Diseases and Infection Prevention, NUTRIM School of Translational Research in Metabolism, Maastricht University Medical Centre+, Maastricht, Netherlands; ^3^Department of Clinical Pharmacy and Pharmacology, Máxima Medical Center, Veldhoven, Netherlands; ^4^Department of Clinical Pharmacy and Toxicology, Maastricht University Medical Centre+, Maastricht, Netherlands; ^5^Department of Medical Microbiology, Infectious Diseases and Infection Prevention, CAPHRI School of Public Health and Primary Care, Maastricht University Medical Centre+, Maastricht, Netherlands

**Keywords:** inflammatory bowel disease, Crohn’s disease, ulcerative colitis, microbiome, pharmacomicrobiomics, drug treatment, drug metabolism

## Abstract

**Background:**

Inflammatory bowel disease (IBD) is a chronic relapsing-remitting disease. An adverse immune reaction toward the intestinal microbiota is involved in the pathophysiology and microbial perturbations are associated with IBD in general and with flares specifically. Although medical drugs are the cornerstone of current treatment, responses vary widely between patients and drugs. The intestinal microbiota can metabolize medical drugs, which may influence IBD drug (non-)response and side effects. Conversely, several drugs can impact the intestinal microbiota and thereby host effects. This review provides a comprehensive overview of current evidence on bidirectional interactions between the microbiota and relevant IBD drugs (pharmacomicrobiomics).

**Methods:**

Electronic literature searches were conducted in PubMed, Web of Science and Cochrane databases to identify relevant publications. Studies reporting on microbiota composition and/or drug metabolism were included.

**Results:**

The intestinal microbiota can both enzymatically activate IBD pro-drugs (e.g., in case of thiopurines), but also inactivate certain drugs (e.g., mesalazine by acetylation *via* N-acetyltransferase 1 and infliximab *via* IgG-degrading enzymes). Aminosalicylates, corticosteroids, thiopurines, calcineurin inhibitors, anti-tumor necrosis factor biologicals and tofacitinib were all reported to alter the intestinal microbiota composition, including changes in microbial diversity and/or relative abundances of various microbial taxa.

**Conclusion:**

Various lines of evidence have shown the ability of the intestinal microbiota to interfere with IBD drugs and vice versa. These interactions can influence treatment response, but well-designed clinical studies and combined *in vivo* and *ex vivo* models are needed to achieve consistent findings and evaluate clinical relevance.

## 1. Introduction

Inflammatory bowel disease (IBD) is a chronic relapsing disease, including Crohn’s disease (CD) and ulcerative colitis (UC). Currently, three in 1,000 people in Western countries and up to one in 1,000 in Asian and South American countries are affected by IBD, of which many experience a substantial disease burden (Ng et al., 2017; Knowles et al., 2018a,b). To date, the pathophysiology is not entirely elucidated, but a genetic predisposition, environmental factors, an aberrant immune reaction against the intestinal microbiota, and intestinal microbial dysbiosis are all assumed to play major roles (Ordás et al., 2012a; Torres et al., 2017; Weingarden and Vaughn, 2017). Although these factors apply to both, UC and CD, clear differences can be observed between these disease phenotypes (Ordás et al., 2012a; Torres et al., 2017), also with regard to microbial dysbiosis (Prosberg et al., 2016; Vich Vila et al., 2018). As compared to healthy individuals, the microbiota of CD patients is characterized by a decrease of fecal and mucosal microbial (species) diversity, decreased temporal stability, and a change in the relative abundance of specific bacterial taxa. Although the exact microbial species associated with CD differ between studies and between fecal samples as compared to mucosal specimens (Ahmed et al., 2016), several more consistent findings include a reduction in the butyrate-producing bacteria *Faecalibacterium prausnitzii*, *Eubacterium rectale*, and *Roseburia intestinalis* and an increase in *Fusobacterium nucleatum* and the facultative aerobic *Enterobacteriaceae* (Willing et al., 2010; Walker et al., 2011; Morgan et al., 2012; Fujimoto et al., 2013; Gevers et al., 2014; Pascal et al., 2017; Galazzo et al., 2019; Clooney et al., 2021).

Microbiota perturbations seem to be less pronounced in UC patients, although a decreased microbial diversity and temporal stability has also been observed in UC patients as compared to healthy controls (Halfvarson et al., 2017; Clooney et al., 2021). Whereas some studies find comparable changes in pediatric IBD as compared to adult patients, including decreased abundances of Actinobacteria and Bacteroidetes and an increased abundance of Proteobacteria, others report some differences (Eindor-Abarbanel et al., 2021). For instance, the ileal mucosa of pediatric CD patients has been shown to harbor higher numbers of *Pseudomonas* species (Wagner et al., 2008), an observation not commonly reported in adult patients.

**FIGURE 1:**
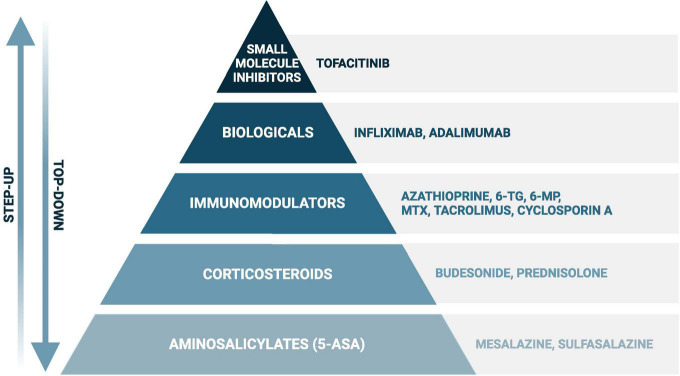
Simplified scheme of the most frequently used drugs in inflammatory bowel disease. When following the “step up” approach, disease exacerbations will first be treated with aminosalicylates (in ulcerative colitis) or corticosteroids, such as prednisolone and budesonide. In case of failure, or to achieve steroid-free remission, immunomodulators, biologicals, and small molecule inhibitors (in ulcerative colitis) will be considered. A certain drug choice strongly depends on several additional factors, such as disease severity and location, and previous treatment history. The “step-up” approach can be found in the recent guidelines of the European Crohn’s and Colitis Organisation, while evidence is rising favoring the “top-down” approach in certain cases with poor prognostic outcome (Harbord et al., 2017; Berg et al., 2019; Torres et al., 2020). Created with BioRender.com.

As no curative treatment for IBD exists, current therapeutic strategies aim to induce and maintain remission, which is more specifically defined as achieving endoscopic healing, absence of disability, and optimal health-related quality of life (Turner et al., 2021). In both, CD and UC, the European Crohn’s and Colitis Organisation (ECCO) recommends local and systemic corticosteroids or biologicals in case of steroid refractive disease to induce remission, and immunomodulators and biologicals to maintain remission. The use of aminosalicylates and small molecule inhibitors to induce and maintain remission are specifically recommended in UC (Torres et al., 2020; Raine et al., 2022). These classes of drugs (Figure 1) all target the adverse immune reaction, but can also lead to moderate to severe side effects, such as myelosuppression by thiopurines (Timmer et al., 2016). Remarkably, treatment non-responses vary between drugs and individuals and occur on average in up to 30–50% of the patients (Baumgart et al., 2008; Duricova et al., 2010; Chande et al., 2014, 2015; McDonald et al., 2014; Patel et al., 2014; Li et al., 2015; Wang Y. et al., 2015; Feuerstein et al., 2016; Meijer et al., 2016; Timmer et al., 2016; Sandborn et al., 2017). For instance, initial response rates for the thiopurine azathioprine range from 40 to 55% in UC and 69–73% in CD, while response rates for methotrexate vary even more in CD, ranging from 19 to 89% (Chande et al., 2014, 2015; McDonald et al., 2014; Timmer et al., 2016). Furthermore, loss of response (i.e., secondary non-response) is not uncommon after initial successful remission induction (Behm and Bickston, 2008).

For some drugs, mechanisms contributing to non-response or side effects are well described, such as antibody formation against anti-tumor necrosis factor (TNF) biologicals or hepatotoxicity by thiopurines based on thiopurine S-methyl transferase (TPMT) induced 6-methylmercaptopurine ribonucleotides (6-MMPR) (Ben-Horin and Chowers, 2011; Chang and Cheon, 2019). In the vast majority, however, even for these drugs, clear explanations are lacking and individual treatment response is difficult to predict (Chande et al., 2015; Timmer et al., 2016; Abbass et al., 2019; Lair-Mehiri et al., 2020; Murray et al., 2020). Identifying the right treatment for the right patient is therefore largely based on trial and error and often leads to loss of time by prescription of non-effective drugs.

Emerging evidence suggests that part of the inter-individual variation in drug response might be attributable to the interactions between drugs and the intestinal microbiome (Zimmermann et al., 2019). The intestinal microbiota, a complex and diverse ecosystem that consists of trillions of microbes mainly belonging to the phyla of Firmicutes, Bacteroidetes and Actinobacteria (Zhernakova et al., 2016), strongly mediates the interaction of the human host and its environment (Lynch and Pedersen, 2016). This includes bidirectional interactions between the gut microbiome and medical drugs, also known as pharmacomicrobiomics (Maier et al., 2018; Zimmermann et al., 2019). This may impact pharmacokinetics, i.e., drug availability and pharmacodynamics, i.e., treatment response and eventual side effects. Drug availability can be influenced directly by interactions with the intestinal microbiota. The duodenal and jejunal microbiota are most relevant for interactions with orally ingested systemic drugs, which are mainly absorbed in the proximal intestine. Examples are prednisolone and azathioprine. The fecal or colonic microbiota is important to investigate interactions with colon-targeting or rectally administered drugs, such as budesonide and mesalazine. These direct interactions can subsequently modulate treatment response and side effects. Besides, the colonic microbiota may also have an indirect effect on systemic drugs. For instance, inactive metabolites of the chemotherapeutic irinotecan, excreted *via* the bile, can be transformed into the active component SN-38G by the colonic microbiota, which leads to mucosal toxicity (Koppel et al., 2017). In addition, the intestinal microbiota can have an indirect impact on drug metabolism by altering host’s metabolism, for instance, *via* bile acid metabolism or other microbial metabolites. This has been shown by microbiota-induced downregulation of the Constitutive Active/Androstane Receptor (CAR) on hepatocytes, which is involved in xenobiotic metabolism (Björkholm et al., 2009; Wahlström et al., 2016). The mechanism of CAR downregulation remains speculative, but might be due to lower levels of CAR activators, including bilirubin, bile acids, and steroid hormones, in the presence of intestinal microbes (Björkholm et al., 2009).

Besides the effect of the microbiota on the fate of drugs, xenobiotics can in turn influence intestinal microbiota composition and function, for instance, by selective growth inhibition (Maier et al., 2018). The intestinal microbial perturbation observed in IBD patients (Ahmed et al., 2016; Santoru et al., 2017) is likely not only an etiological factor contributing to the disease, but may also be partly the result of perturbations caused by inflammatory processes and medication use. For example, the TNF-inhibitor infliximab has been shown to increase fecal microbial diversity, lower microbial diversity in mucosal biopsies, and increase levels of short-chain fatty acid producing bacteria (e.g., *Roseburia, Lachnospira*, and *Blautia*), while decreasing levels of opportunistic pathogenic bacteria (*Fusobacterium*, *Enterobacter*, *Escherichia*) (Dovrolis et al., 2020; Zhuang et al., 2020). The use of thiopurines has been associated with a reduced fecal microbial diversity (Wills et al., 2014). Hereby, IBD drugs may indirectly act on intestinal inflammation *via* altering the intestinal microbiota composition and function.

The aim of this extensive narrative review is to elucidate the evidence and clinical relevance of bidirectional drug-microbiota interactions (pharmacomicrobiomics) in IBD. To ensure the inclusion of relevant therapeutics, this review follows the treatment recommendations of the ECCO for CD and UC (Harbord et al., 2017; Torres et al., 2020). Therefore, this review will cover the following drugs: aminosalicylates, corticosteroids, immunomodulators, biologicals, and small molecule inhibitors (Figure 1). To achieve a comprehensive overview, we included *in vitro*, animal and human studies investigating the impact of IBD drugs on the intestinal microbiota or vice versa.

We are aware of the recent changes in the microbial taxonomic nomenclature, as summarized by Oren and Garrity (2021). However, for the present review, we decided to use the previous nomenclature to maintain consistency with the reviewed articles and to facilitate readability.

## 2. Aminosalicylates

Sulfasalazine, the first aminosalicylate on the market, consists of the active compound 5-aminosalicylic acid (5-ASA; mesalazine) and sulfapyridine joined by an azo bond. Many other 5-ASA formulations, for instance olsalazine and 5-ASA have been developed since and are often better tolerated due to the lack of sulfapyridine-related side effects (D’Haens and van Bodegraven, 2004; Campregher and Gasche, 2011; Harbord et al., 2017).

Although the exact mode of action of 5-ASA formulations has not been completely elucidated, several mechanisms have been described. The main anti-inflammatory action is proposed to be mediated by binding and activation of peroxisome proliferator-activated receptor (PPAR)-γ (Iacucci et al., 2010; Annese et al., 2012; Berends et al., 2019). This nuclear binding protein attenuates the transcription pathways of nuclear factor-kappa-light-chain-enhancer of activated B cells (NF-κB), activator protein-1 (AP-1), signal transducer and activator of transcription (STAT), and nuclear factor-activated T-cell (NFAT). Thereby, it inhibits the production of pro-inflammatory mediators, such as interleukin (IL) 1β, cyclooxigenase-2, IL-6, IL-8, TNF-α, interferon (IFN)-γ, inducible nitric oxide synthase, and chemokines. In macrophages, PPAR-γ stimulation further promotes the differentiation and conversion of pro-inflammatory M1 into regulatory M2 phenotypes (Annese et al., 2012). Moreover, 5-ASA is thought to be a potent anti-oxidant and free-radical scavenger (Iacucci et al., 2010). Furthermore, although sulfasalazine is often considered to be a prodrug for site-specific delivery of its active component mesalazine, this parent compound has immunopharmacological properties of its own by inhibiting the binding of TNF-α to its cell membrane receptor (Shanahan et al., 1990).

### 2.1. Impact of the intestinal microbiota on aminosalicylate metabolism

Besides drug metabolism in endothelial and liver cells, the intestinal microbiota is also able to directly interfere with aminosalicylates. It is well known that intestinal bacteria are essential for the activation of the orally administered pro-drug sulfasalazine, cleaving the azo bond by bacterial azoreductases. Azoreductases are present in several intestinal bacterial species, such as *Pseudomonas aeruginosa*, *Enterococcus faecalis*, *Escherichia coli*, *Bacillus subtilis*, and *Rhodobacter sphaeroides* (Ryan, 2017). Inter-individual variations in microbiota composition might thus affect sulfasalazine drug metabolism. This is further underscored by a human *ex vivo* study, which showed that fecal cultures from different individuals resulted in different sulfasalazine degradation rates (Supplementary Table 1; Sousa et al., 2014).

5-aminosalicylic acid (does not require bacterial activation. However, N-acetyltransferase 1 in intestinal epithelial cells can inactivate 5-ASA into N-acetyl-5-ASA (Allgayer et al., 1989). This same enzymatic inactivation has also been shown for several intestinal bacteria (Deloménie et al., 2001). Studies on *ex vivo* fecal cultures of IBD patients, and *in vitro* cultures of ten out of 41 single intestinal bacteria could detect varying amounts of acetylated 5-ASA, showing the potential of the intestinal microbiota to metabolize and inactivate 5-ASA as described for human mucosal cells (Allgayer et al., 1989; van Hogezand et al., 1992; Deloménie et al., 2001). Individual differences in microbiota composition could therefore contribute to differences in degradation rates and treatment efficacy, but such studies are lacking. Future research should characterize the fecal microbiota of well-phenotyped UC patients and assess the capacity of the individual fecal microbiota to degrade 5-ASA prior to start of patient treatment. The outcomes can then be linked to patients’ treatment response. Following, individual treatment response may be improved by microbiota-adjusted individual dosing or switching of drug class. 5-ASA dose adjustments may allow the patient to continue with mesalazine treatment instead of switching to a drug with higher risks of adverse effects. Additional research needs to evaluate the safety of higher 5-ASA doses.

### 2.2. Impact of aminosalicylates on the intestinal microbiota

In addition to the effect of microbes on 5-ASA or sulfasalazine, there is some evidence that 5-ASA formulations in turn influence intestinal microbial function and composition. A combined *in vitro* and *ex vivo* study showed that 5-ASA reduces the capacity of intestinal bacterial biofilm or microcolony formation by enzymatic inhibition of polyphosphate kinase (Dahl et al., 2017). This inhibition leads to lower levels of bacterial polyphosphate, which is a virulence factor and involved in biofilm formation, inflammatory oxidant resistance, and host macrophage manipulation (Dahl et al., 2017; Roewe et al., 2020). Although sulfasalazine is not highly recommended in the treatment of UC, due to side effects (Harbord et al., 2017), the antibiotic activity of the sulfonamide moiety, sulfapyridine, may be of interest. The antibiotic activity of sulfonamides interferes with *de novo* folate synthesis and can affect coliform bacteria, including *E. coli* (Zimmermann and Curtis, 2019). Already in 1974, a study reported decreased abundances of Enterobacteria, aerobic, and non-sporing anaerobic bacteria in fecal cultures from IBD patients using sulfasalazine (West et al., 1974). To our knowledge, potential complementary anti-inflammatory effects of sulfapyridine due to subsequent microbiota alterations have not yet been studied in IBD patients.

In a cross-sectional study in UC patients, Olaisen et al. (2019) found a positive correlation between mucosal 5-ASA concentrations and mucosal bacterial diversity. They also found a positive correlation with the abundance of *F. prausnitzii*, *Blautia*, and *Bacteroides*, bacterial taxa known for their beneficial potential and concluded that 5-ASA had a beneficial impact on mucosal bacterial composition with regard to UC disease activity (Olaisen et al., 2019).

Several studies investigated the effect of 5-ASA or sulfasalazine on shifts in intestinal microbiota composition in IBD. An *ex vivo* study with human fecal cultures found a decreased abundance of *Eggerthella lenta* and an increase in *Bacteroides vulgatus* and *Parabacteroides distasonis* after 24 h incubation with sulfasalazine (Li et al., 2020). Moreover, 5-ASA has been shown to inhibit the growth of *E. coli* as well as several *Campylobacter concisus* strains, while promoting the growth of other *C. concisus* strains *in vitro* (Liu et al., 2017). Previously, increased colonization by *E. coli* and especially adherent invasive *E. coli* has been associated with active CD (Ahmed et al., 2016). Therefore, growth inhibition observed for 5-ASA seems beneficial in CD. Longitudinal studies investigating fecal samples of IBD and irritable bowel syndrome patients before and after 5-ASA treatment reported inconsistent results with respect to the observed bacterial and fungal alterations (Supplementary Table 2; Andrews et al., 2011; Morgan et al., 2012; Imhann et al., 2018; Schirmer et al., 2018; Vich Vila et al., 2018; Jun et al., 2020; Wang et al., 2021), which might be due to differences in microbiota composition between patient (sub-)groups. Therefore, future longitudinal cohort studies or randomized controlled trials taking into account various disease phenotypes and environmental factors might be of added value to generate more consistent findings and increase our understanding on the potential 5-ASA-mediated host-microbe interactions.

## 3. Corticosteroids

The most commonly used corticosteroids for IBD treatment are prednisolone and budesonide (Harbord et al., 2017; Torres et al., 2020). Budesonide acts locally and can be administered orally in delayed release formulations or rectally and undergoes extensive pre-systemic elimination because of CYP3A4-mediated metabolism in the intestinal epithelium and by the hepatic first pass effect (Schwab and Klotz, 2001). Oral systemic corticosteroids, such as prednisolone, have a bioavailability of 60–100% and are partially bound to plasma molecules, such as globulin and albumin, and are mainly metabolized by the hepatic CYP3A subfamily of cytochrome P450 (Czock et al., 2005). Corticosteroids are highly lipophilic compounds, of which the soluble form can enter cell membranes by passive diffusion and interact with the nuclear glucocorticoid receptor (GR). After subsequent conformational changes, the corticosteroid-GR complex translocates to the nucleus and induces gene transcription by binding DNA sequences known as glucocorticoid responsive elements (GRE) in the promotor region of target genes (Bruscoli et al., 2021). Hereby, these drugs are able to control the expression of many genes, such as pro-inflammatory mediators and transcription factors (e.g., NF-κB and AP-1), thereby inhibiting the expression of pro-inflammatory cytokines by macrophages, dendritic cells, and T-lymphocytes. Corticosteroids further inhibit inflammation by inducing regulatory dendritic cells and anti-inflammatory M2 macrophages (Dubois-Camacho et al., 2017) and even more anti-inflammatory mechanisms have been described, such as reduced blood vessel proliferation and vasodilatation and decreased clonal expansion of B- and T-lymphocytes (Derijks et al., 2018).

To date, little is known about the mechanisms underlying corticosteroid non-response. Several potential mechanisms have been suggested, including a reduced number of GRs binding to the DNA, higher abundances of the non-steroid binding isoform GR-β, IL-2 and IL-4-promoted steroid resistance, and multi-drug-resistance (*MDR*) 1 gene polymorphisms (Creed and Probert, 2007). *MDR* encodes for the P-glycoprotein (P-gp) 170 drug efflux pump, which excretes glucocorticoids. In addition, non-genetic mechanisms may contribute to corticosteroid non-response. For instance, epigenetic histone- and DNA-modifying enzymes can alter GR-related gene expression, while non-coding RNA prevents the binding of GR to GREs, thereby preventing target gene expression (Bartlett et al., 2019).

### 3.1. Impact of the intestinal microbiota on corticosteroid metabolism

A recent *in vitro* culture study showed that a variety of intestinal bacterial species and strains were able to degrade budesonide and the pro-drug prednisone, such as *Bacteroides eggerthii*, *Bacteroides fragilis*, and an undetermined *Clostridium* species, but to different extents (Zimmermann et al., 2019). Budesonide as well as the active drug prednisolone were completely degraded in *ex vivo* fecal cultures after 7 and 2.5 h, respectively (Yadav et al., 2013). A potential degradation mechanism is cleavage of the steroid side chain by bacterial steroid-12,20-desmolase, although this enzyme is only expressed by few intestinal bacteria (Morris and Ridlon, 2017). Another *in vitro* study confirmed this mechanism by detecting 11-keto-1,4-androstene-3,17-dione and 11β-hydroxy-1,4-androstene-3,17-dione as products of *Clostridium scindens* metabolism of prednisone and prednisolone, respectively (Supplementary Table 1; Ly et al., 2020). It has been proposed that bacterial steroid-12,20-desmolase-mediated steroid metabolism provides carbon and energy for bacterial growth (Morris and Ridlon, 2017). Further *ex vivo* fecal culture studies are needed to elucidate additional microbial degradation pathways of budesonide as well as to explore degradation rates in IBD patients to estimate the relevance in relation to corticosteroid non-response. For prednisolone, a similar approach can be valuable, using microbial consortia that represent key microbial taxa of the proximal intestine.

### 3.2. Impact of corticosteroids on the intestinal microbiota

Few studies have reported on the effects of budesonide and prednisolone on intestinal microbial growth and microbiota composition. A large *in vitro* study by Maier et al. (2018) did not detect any alterations in microbial growth when screening 40 intestinal bacterial strains. Two animal studies on mucosal biopsies of IBD dogs and fecal samples from healthy mice found alterations in the microbial composition upon prednisone or prednisolone administration, respectively, whereas a study in healthy dogs did not find microbiota alterations upon prednisolone administration (Igarashi et al., 2014; Tourret et al., 2017; Atherly et al., 2019). A recent study comparing *ex vivo* human fecal cultures from five individual donors incubated with methylprednisolone, only found a decreased abundance of *Bifidobacterium longum* (Li et al., 2020). Budesonide, administered to patients with microscopic colitis resulted in increased abundances of *Ruminococcaceae ge* and *Ruminococcus* 2, but decreases of *Faecalibacterium* abundance. In addition, bacterial diversity has been increased (Rindom Krogsgaard et al., 2019). In this case, the diversity describes the intestinal bacterial diversity within one patient, which is generally higher in healthy individuals and lower in intestinal inflammation (Huttenhower et al., 2012; Ahmed et al., 2016; Rindom Krogsgaard et al., 2019). Finally, a large study in pediatric UC patients reported many taxa to be differentially abundant upon unspecified corticosteroid treatment (Schirmer et al., 2018). In addition, the affected taxa differed when comparing responders and non-responders. Together, the above findings indicate that corticosteroids may affect the microbiota composition in IBD patients (Supplementary Table 2). However, the findings are rather incomparable due to different study populations (i.e., different animals, healthy people and IBD patients), study designs (i.e., cross-sectional studies analyzing feces or mucosal biopsies, bacterial strain culture), and methods (i.e., marker gene sequencing, optical density measurements). Therefore, it is yet impossible to draw firm conclusions with regard to the impact on gut health or inflammation in IBD. Well-designed longitudinal cohort studies, focusing on individual drugs and clearly differentiated IBD patient (sub)groups are needed to determine solid effects of specific corticosteroids in well-defined IBD patient groups.

## 4. Immunomodulators

### 4.1. Thiopurines

Thiopurines used for the treatment of IBD include the pro-drugs azathioprine (AZA) and 6-mercaptopurine (6-MP). In addition, 6-thioguanine (6-TG) is considered as rescue thiopurine in defined clinical situations, due to potential hepatotoxicity of 6-TG (de Boer et al., 2006). The active metabolites of thiopurine drugs are the 6-thioguanine nucleotides (6-TGNs), which have been described to have various inhibitory effects on inflammatory mechanisms. For instance, 6-TGN can inhibit Ras-related C3 botulinum toxin substrate (Rac) 1, a GTPase of the CD28 downstream signaling cascade, which leads to T-lymphocyte apoptosis (Derijks et al., 2018). 6-TGNs also inhibits the expression of inflammatory genes in T-lymphocytes (Hvas et al., 2018). Further, the thiopurine metabolite 6-MMPR may inhibit *de novo* purine synthesis, consequently inhibiting proliferation (Derijks et al., 2018).

Genetic variations of different liver enzymes, such as TPMT, have been shown to be implicated in thiopurine non-response and/or side effects. TPMT inactivates different thiopurine drug metabolites. In case of single nucleotide polymorphisms, enzyme activity is reduced or impaired and associated with myelosuppression. Therefore, *TPMT* genotyping and subsequent dose adjustment is now recommended before starting thiopurine therapy, to prevent myelosuppression (Roberts and Barclay, 2012; Coenen et al., 2015; Marinaki and Arenas-Hernandez, 2020). Additionally, *Nudix hydrolase* (*NUDT) 15* and *Fat mass and obesity associated protein* (*FTO)*, which catalyze subsequent conversions of 6-MP, are also associated with myelosuppression, occurring in up to 40% of the thiopurine users (Candy et al., 1995; Panés et al., 2013; Chang and Cheon, 2019). Furthermore, Glutathione S-transferase (GST)-M1 deletion has been associated with lower levels of TGNs in IBD patients and reduced treatment response (Stocco et al., 2014). Normally, GST family enzymes convert AZA into 6-MP, with isoforms GST-A1, A2, and M1 being the main contributors (Roberts and Barclay, 2012). Still, the majority of non-responders remain unexplained. Since azathioprine, 6-MP and 6-TG are administered orally, they may interact with the intestinal microbiota prior to resorption.

#### 4.1.1. Impact of the intestinal microbiota on thiopurine metabolism

**FIGURE 2:**
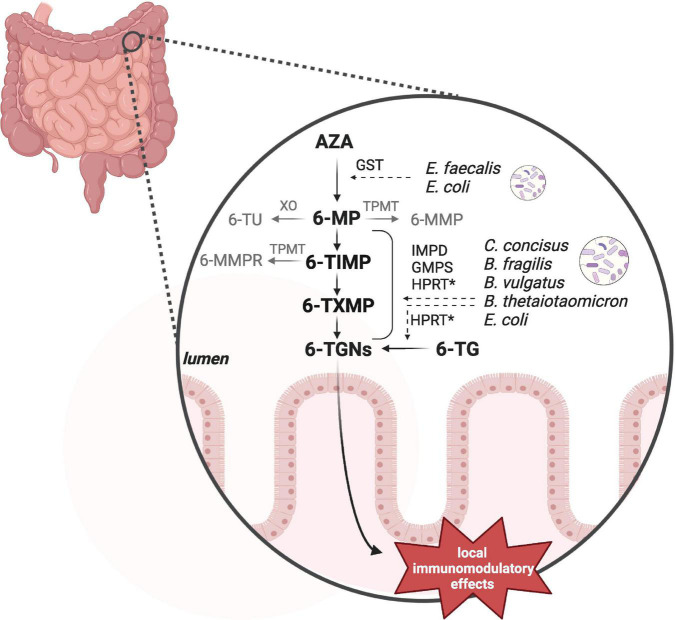
Bacterial thiopurine conversion. Azathioprine (AZA) and 6-mercaptopurine (6-MP) can be converted by microbial enzymes into the anti-inflammatory 6-tioguanine nucleotides (6-TGNs). *In vitro* studies observed different enzymatic processes and the responsible genes by a number of intestinal bacteria, including *Enterococcus faecalis*, *Escherichia coli*, *Campylobacter concisus*, *Bacteroides fragilis*, *Bacteroides vulgatus*, and *Bacteroides thetaiotaomicron*. Not all of these species contained all of the required enzymes to individually catalyze the complete pathway. GST, glutathione S-transferase; HPRT, hypoxanthine guanine phosphoribosyl transferase; IMPD, inosine monophosphate dehydrogenase; GMPS, guanosine monophosphate synthetase. *Hprt* has not been detected in *C. concisus* by Liu et al. (2017). Created with BioRender.com.

Recent *in vitro* research found that some intestinal bacterial strains contain the enzymes to catalyze the conversions from AZA into the active metabolites 6-TGNs (Movva et al., 2016; Liu et al., 2017). Some of these commensal bacteria have earlier been shown to be more abundant in IBD when compared to healthy subjects, including *C. concisus*, *E. coli* and *B. fragilis* (Ahmed et al., 2016). However, not every strain tested contained all the enzymes required. For instance, *B. fragilis* ATCC 25285 lacked GST, which catalyzes AZA into 6-MP (Liu et al., 2017). This means that a combination of bacterial species is needed to convert AZA into 6-TGNs (Figure 2, Supplementary Table 1). A recent murine study confirmed the microbial conversion of 6-TG into 6-TGNs and to a lesser extent of 6-MP into 6-TGNs (Oancea et al., 2017). This study further investigated non-systemic effects of 6-TGNs in *Hypoxanthine guanine phosphoribosyl transferase*^–/–^ mice, which are not able to catalyze 6-TG into 6-TGNs. They noticed that exclusive bacterial conversion into 6-TGNs could ameliorate dextran sodium sulfate-induced colitis (Oancea et al., 2017). Together, these findings indicate that the intestinal microbiome can contribute to enhancement of the local anti-inflammatory effect *via* 6-TGNs. In future, this may provide the opportunity of local thiopurine treatment in IBD, which would reduce the side effects associated with systemic thiopurine treatment, such myelosuppression and liver toxicity (Florin et al., 2018). Therefore, a clinical non-inferiority trial is warranted to study the potential of exclusive microbiota-aided local thiopurine therapy in IBD patients and further the extent of interindividual variation based on a patient’s microbiota composition.

#### 4.1.2. Impact of thiopurines on the intestinal microbiota

Thiopurines by themselves can affect the microbiota composition. In a small study, our group found a lower bacterial diversity in fecal samples of thiopurine-treated CD and UC patients (Wills et al., 2014). An *ex vivo* study by Swidsinski et al. showed higher numbers of mucosa associated bacteria in biopsies of AZA treated patients compared to healthy and largely untreated IBD controls, and a higher amenability of mucosal bacteria when compared to otherwise treated IBD patients. This effect was accompanied by a lower mucosal leukocyte count, which may be explained by a direct effect of AZA on the immune response, or by a thiopurine-induced bacteriostatic effect that reduces the need for mucosal invasion of leukocytes (Swidsinski et al., 2007; Liu et al., 2017). Further evidence on the impact of thiopurines on the intestinal microbiota originates from animal and *in vitro* data. In healthy wild type mice, Oancea et al. observed a reduction in Bacteroidetes and an increase in Firmicutes abundance upon 28 days 6-TG administration (Oancea et al., 2017). Since reduced levels of Firmicutes have been observed in IBD (Ahmed et al., 2016), 6-TG administration may help to change the patient’s microbiota toward a “healthier” composition. However, we could not identify studies investigating this effect in IBD patients using 6-TG. *In vitro*, AZA and 6-MP were found to inhibit (dose-dependently) the growth of several bacterial strains, including *C. concisus* strains, *B. fragilis*, *B. vulgatus*, *E. lenta*, and *E. coli* (Supplementary Table 2; Liu et al., 2017; Maier et al., 2018), which might explain at least partially the above described reduced bacterial diversity in fecal samples (Wills et al., 2014).

More mechanistic *in vitro* studies showed that bacterial 6-TG, produced by *Erwinia* species, could inhibit growth of *E. coli* and *Salmonella typhimurium* (Wensing et al., 2014), and an inhibitory effect of AZA and 6-MP on bacterial cyclic-di-GMP (Antoniani et al., 2013; Migliore et al., 2018). This molecule is produced by Gram-negative bacteria and is important in biofilm formation. In adherent invasive *E. coli* (AIEC), 6-MP further impairs motility and virulence, potentially *via* downstream pathways of c-di-GMP, which normally induces the formation of pili and fimbriae. Since AIEC was shown to play a role in CD (Ahmed et al., 2016), this off-target effect of 6-MP may contribute to reduce inflammation. Similar off-target anti-inflammatory effects *via* bacterial c-di-GMP inhibition may also be applicable to sulfasalazine, because of structural similarity of the sulfapyridine to sulfathiazole, which has also been described to prevent the formation of c-di-GMP (Antoniani et al., 2013). To better understand the spectrum of bactericidal effects by thiopurines and their potential benefit to reduce intestinal inflammation, future studies should specifically focus on bacterial growth inhibition by thiopurines and the pro- or anti-inflammatory potential of identified target bacteria.

### 4.2. Methotrexate

Methotrexate (MTX) is an analog of dihydrofolate (DHF), which is required for *de novo* purine and pyrimidine synthesis and subsequent DNA synthesis and cell proliferation (Roberts and Barclay, 2012). The mechanisms of action of MTX are not completely elucidated and are considered to be dose-dependent. MTX can be resorbed as active drug by host cells and can be polyglutamylated (MTX-PG), which increases its binding affinity for target enzymes (Sayers et al., 2018) and accumulate in the cell, allowing a once-weekly drug treatment (Grim et al., 2003). In contrast to cancer treatment, MTX is used in lower doses for the treatment of IBD (Patel et al., 2014; Wang Y. et al., 2015; Howard et al., 2016). In low dose treatment, MTX was found to block the enzyme 5-aminoimidazole-4-carboxamide ribonucleotide formyltransferase/Inosine monophosphate cyclohydrolase (ATIC), resulting in intracellular adenosine accumulation and subsequent excretion (Cronstein et al., 1993; Roberts and Barclay, 2012). Extracellular adenosine then binds to G-protein-coupled adenosine receptors on target cells. For instance, in T-cells, binding to the A_2a_ receptor leads to a downstream cascade involving cAMP and protein kinase A, and an anti-inflammatory phenotype (Cronstein et al., 1993; Cronstein and Sitkovsky, 2017). In macrophages, A_2a_ stimulation leads to reduction of pro-inflammatory cytokines, such as TNF-α and IFN-γ (Cronstein and Sitkovsky, 2017). Other potential mechanisms of action include inhibition of DHF reductase resulting in nitric oxide synthase uncoupling and the activation of JUN N-terminal kinases, increased expression of long intergenic non-coding RNA p21 leading to diminished immune and inflammatory reactions, and suppression of NF-κB activity and of signal transducer and activator of transcription (STAT) proteins by receptor- associated Janus kinases (JAKs) (Hvas et al., 2018; Cronstein and Aune, 2020).

To date, only few studies address mechanisms of non-response to MTX in IBD. For example, a decreased efficacy of methotrexate was found in patients with high *MDR* expression in peripheral lymphocytes and epithelial cells (Farrell et al., 2000). *MDR* encodes for the P-gp 170 drug efflux pump, which excretes glucocorticoids and methotrexate (Sayers et al., 2018). More studies have been conducted exploring mechanisms of MTX toxicity, such as the homozygous Methylenetetrahydrofolate reductase gene 1298C variant which was associated with increased toxicity and side effects, such as nausea and vomiting (Herrlinger et al., 2005).

#### 4.2.1. Impact of the intestinal microbiota on methotrexate metabolism

**FIGURE 3:**
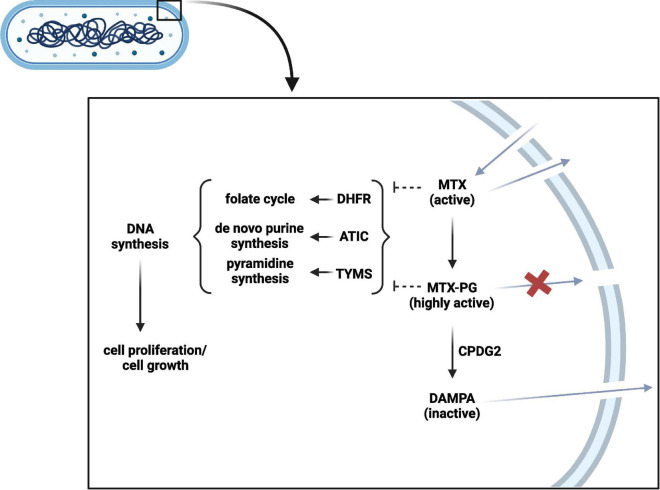
Bacterial methotrexate (MTX) metabolism and interaction. MTX can enter the bacterial cell and can be polyglutamated into highly active MTX-PG. CPDG2-like enzymes can in turn inactivate MTX-PG into DAMPA. In contrast to MTX-PG, MTX and DAMPA can leave the bacterial cell. MTX and MTX-PG can interfere with DHFR, ATIC, and TYMS to subsequently inhibit DNA synthesis and bacterial growth. PG, polyglutamate; CPDG2, carboxypeptidase glutamate 2; DAMPA, 2,4-diamino-N-10-methylpteroic acid; DHFR, dihydrofolate reductase; ATIC, 5-aminoimidazole-4-carboxamide ribonucleotide formyltransferase/Inosine monophosphate cyclohydrolase; TYMS, thymidylate synthetase. Created with BioRender.com.

In contrast to oral administration for the treatment of rheumatic diseases, MTX is preferentially administered parenterally in the treatment of Crohn’s disease (Torres et al., 2020). Still, interactions with the microbiota are relevant, as MTX can re-enter the intestinal lumen due to the enterohepatic circulation and should be considered when evaluating treatment success (Figure 3; Derijks et al., 2018). Already in 1975, an *in vitro* bacterial culture study showed that *Lactobacillus casei* can resorb MTX and low glutamylated folates (Shane and Stokstad, 1975). As several bacteria depend on folate uptake for growth, it is likely that they can also interact with MTX. A recent conference abstract confirmed that several bacteria are able to interfere with MTX by resorbing and converting it into MTX-PG, which prevents cellular excretion and increases its binding affinity to target enzymes, such as ATIC (Supplementary Table 1; Nayak et al., 2017; Sayers et al., 2018). In addition, bacteria can convert MTX-PG into the inactive metabolite 2,4-diamino-N-10-methylpteroic acid (DAMPA) (Levy and Goldman, 1967; Letertre et al., 2020). Examples of bacterial enzymes catalyzing this latter reaction are the *E. coli* p-aminobenzoyl-glutamate hydrolase (PGH) and the *Pseudomonas* carboxypeptidase glutamate 2 (CPDG2) (Larimer et al., 2014). Today, CPDG2 administration is recommended for patients with MTX-PG intoxications (Buchen et al., 2005; Ramsey et al., 2018). Yet, it remains unclear whether the amount of bacterial MTX uptake and metabolism plays a role in treatment non-response by forming a reservoir of MTX that cannot reach the host circulation or by MTX inactivation. Another interesting finding was made by a clinical study in ten rheumatoid arthritis patients, which found a substantial increase in accumulation of long chain MTX-PG in erythrocytes after switching from oral to parenteral administration, which was further correlated with treatment response (Dervieux et al., 2010). This allows the hypothesis that intestinal microbes may alter MTX in suchlike that host cell MTX polyglutamylation, and thereby probably its action, is impaired. However, the causality and underlying mechanisms remain to be elucidated. Altogether, the current findings demonstrate that the intestinal microbiota can interfere with MTX. Future combined *ex vivo* and clinical research is needed to clarify the potential relation with treatment response and whether microbiota perturbations of IBD patients as well as conventional dosages in IBD are relevant in this. Ideally, the microbiota of fecal donations should be investigated on their ability to resorb and metabolize MTX prior the start of patient treatment. *Ex vivo* results can then be linked to clinical treatment response.

#### 4.2.2. Impact of methotrexate on the intestinal microbiota

The impact of MTX on the intestinal microbiota composition and function is yet unclear. Similar to humans, bacteria make use of folate pathway products for *de novo* purines and pyrimidine synthesis and subsequent DNA synthesis, and cell proliferation. Although the pathways are very similar, the protein sequence similarity of for example dihydrofolate reductase (DHFR), which converts folates into its active form, is rather low and differs largely between bacterial taxa (Liu et al., 2013). However, a study on ATIC of *Staphylococcus lugdunensis* suggested that the active site of the bacterial ATIC is functionally comparable to the human enzyme (Verma et al., 2017). Therefore, it seems likely that MTX can serve as substrate to DHFR and ATIC in several microbial taxa and inhibit microbial growth. In line with these findings, *in vitro* studies showed (dose-dependent) growth inhibition of a number of bacterial species upon MTX exposure, such as *Ruminococcus gnavus*, *B. fragilis*, *Clostridium perfringens*, and *Mycobacterium avium* (Greenstein et al., 2007; Maier et al., 2018; Nayak et al., 2019). In contrast, other species appeared resistant, such as *F. prausnitzii*, *E. coli*, and *Eggerthella lenta* (Maier et al., 2018; Nayak et al., 2019), which may be due to the presence of enzymes, such as the multi-drug efflux pump TolC (Kopytek et al., 2000). On the phylum level, Firmicutes tend to be more resistant to MTX than Bacteroidetes (Nayak et al., 2019).

In mice, MTX treatment resulted in a reduced bacterial diversity, decreased relative abundance of *B. fragilis*, Bacteroidales and Ruminococcaceae, and an increased abundance of Lachnospiraceae (Zhou et al., 2018). In line with these findings, other murine models (with and without transplantation of fecal microbiota of a healthy human donor), showed an increase in Firmicutes and Proteobacteria and a decrease in Bacteroidetes and Verrucomicrobia (Ahmed et al., 2016; Nayak et al., 2019; Letertre et al., 2020). Studies investigating alterations in the fecal microbiota of rheumatoid arthritis patients, IBD patients, and fecal cultures from healthy people found different alterations of bacterial abundances before and after MTX treatment initiation, including a decrease in Bacteroides in several studies (Zhang et al., 2015; Imhann et al., 2018; Nayak et al., 2019; Li et al., 2020). Together, these different studies show that MTX favors growth and abundance of Firmicutes, which are generally found to be decreased in IBD patients (Ahmed et al., 2016). This might indicate a direct or indirect MTX-induced conversion toward a “healthier” microbiota composition. Also, growth inhibition of *R. gnavus*, *B. fragilis*, and *M. avium* and growth resistance of *F. prausnitzii* and *E. coli* may be favorable for gut health, as *R. gnavus*, *B. fragilis*, and *M. avium* exert pro-inflammatory characteristics and are associated with IBD, whereas *F. prausnitzii* has anti-inflammatory characteristics and has been found to be decreased in IBD (Greenstein et al., 2007; Ahmed et al., 2016; Becker et al., 2021). The physiological impact of *E. coli* is highly strain dependent and therefore difficult to predict on species level, since the pro-inflammatory AIEC has been associated with IBD, whereas the probiotic strain Nissle 1917 was shown to improve intestinal barrier function (Bischoff et al., 2014; Ahmed et al., 2016). However, the observed MTX-induced decreased bacterial diversity in mice seems rather unfavorable (Zhou et al., 2018), since a lower diversity is associated with IBD (Ahmed et al., 2016). In addition, the different results obtained from human studies make a definitive conclusion difficult.

A combined *in vitro* and *in vivo* study on the anti-folate antibiotic drug sulfamethoxazole could demonstrate a drug-induced alteration in *E. coli* metabolism. Upon exposure, *E. coli* upregulated the production of potent so-called colipterin antioxidants, which subsequently lead to the production of anti-inflammatory IL-10 and amelioration of dextran sodium sulfate-induced colitis in mice (Park et al., 2020). Further research is needed to explore whether this effect also applies to MTX.

In summary, research showed that MTX interactions can directly and indirectly alter the growth of several intestinal bacteria, thereby influencing the microbiota composition (Supplementary Table 2). Furthermore, anti-folates can also alter bacterial function, stimulating anti-inflammatory response (Park et al., 2020). To our knowledge, only one study by Imhann et al. (2018) investigated the effect of MTX on the microbiota in IBD patients. Future studies should focus on the relevance of MTX-induced microbiota alterations in treatment response. For example, this may be approached by fecal transplantation from IBD patients on MTX to a colitis mouse model to evaluate potential anti-inflammatory effects. Additionally, a local anti-inflammatory effect as shown for sulfamethoxazole, should be investigated for MTX. This may be a promising complementary therapy to reduce inflammation locally, thereby reducing systemic drug-related side effects. To this end, a colitis mouse model may be used and administered with colonic release capsules containing MTX.

### 4.3. Calcineurin inhibitors

Calcineurin is a eukaryotic calcium- and calmodulin-dependent protein serine/threonine phosphatase, which is involved in several intracellular pathways, amongst others related to cell proliferation and differentiation (Rusnak and Mertz, 2000). Calcineurin activates transcription factors, such as NFAT, which stimulates the production of pro-inflammatory cytokines (e.g., IL-2, IL-4, and IL-17) and activates T-lymphocytes (van Dieren et al., 2006). Calcineurin inhibitors, such as cyclosporine A (or ciclosporin) and tacrolimus, inhibit calcineurin and thereby T-cell activation. Furthermore, they inhibit activation of c-Jun N-terminal kinase, p38 and NF-κB, and potentially induce the expression of the regulatory protein Transforming growth factor-β1 (Barbarino et al., 2013).

Orally administered tacrolimus and cyclosporine A enter the intestinal epithelial cell *via* yet unknown active transporters and *via* passive diffusion (Barbarino et al., 2013; Tron et al., 2019). A large part is then metabolized into less potent or inactive metabolites, while the remaining parent drug can leave the enterocyte and enters the circulation. The amount of active drug is further limited by binding to erythrocytes and by liver enzyme degradation, mainly by CYP3A4 and CYP3A5. The most abundant but less active metabolites of tacrolimus and cyclosporine A are 13-O-Demethyl-tacrolimus and AM1, AM9, and AM4N, respectively.

Overall, the bioavailability is about 25% with large interindividual differences (Barbarino et al., 2013). Drug bioavailability of both, tacrolimus and cyclosporin A, strongly depends on liver enzyme activity and the abundance of apical P-gp drug efflux pumps in enterocytes. The higher the P-gp levels in enterocytes, the more drug is prevented from entering the systemic circulation and reaching the drug target. Instead, drug concentrations can increase in the intestinal lumen (Lown et al., 1997; Barbarino et al., 2013). In UC, higher remission rates have been found in TT (thymine-thymine) genotypes of *ABCB1* encoding P-gp for SNPs rs1045642, rs2032582, and rs1128503 (Herrlinger et al., 2011). The TT variant of rs1045642 was associated with a lower P-gp expression level in enterocytes. Thereby, tacrolimus was suggested to be accumulating at the mucosal site and having a larger local effect (Herrlinger et al., 2011). In contrast, mechanisms of non-response are hardly studied.

#### 4.3.1. Impact of the microbiota on calcineurin inhibitor metabolism

So far, only a few *in vitro* studies investigated the effect of intestinal microbes on calcineurin inhibitors. Guo et al. (2019) showed that several bacterial taxa, including *Bacteroides* species, *Clostridium* species, and *R. gnavus*, as well as fecal cultures of two healthy individuals were able to convert tacrolimus into the metabolites, listed as M1, a less potent immunosuppressant, and M2, a tautomer of M1 with unknown potency. Of note, these bacterial M1 and M2 were structurally different from the above described human liver metabolites M1 and M2 (Iwasaki, 2007; Guo et al., 2019). A study by Wang et al. showed that human fecal cultures were able to metabolize cyclosporine A with a *t*_1/2_ = 422.56 minutes (Supplementary Table 1; Wang J. et al., 2015). Although the evidence on microbial calcineurin inhibitor metabolism is scarce, clear leads have been found which point toward potential microbial contribution to treatment non-response (Wang J. et al., 2015; Guo et al., 2019). Future *ex vivo* and mechanistic studies are requested to clarify inter-individual differences in the fraction and velocity of calcineurin inhibitor metabolism and to investigate the pharmacodynamic properties of the resulting metabolites.

#### 4.3.2. Impact of calcineurin inhibitors on the intestinal microbiota

Based on the various physiological functions of calcineurin and related pathways, especially in cell proliferation and differentiation, eukaryotic microbes can be affected by calcineurin inhibitors (Park et al., 2019). Cyclosporine A and tacrolimus were both found to be effective antifungal agents (Rusnak and Mertz, 2000; Guo et al., 2019). Given the absence of calcineurin in prokaryotes, interaction of calcineurin inhibitors with bacteria seems unlikely, but may still occur *via* other targets. Bacterial cell entry is possible due to the high lipophilicity of calcineurin inhibitors (Barbarino et al., 2013). On different bacterial cultures, cyclosporine A was indeed shown to have an inhibitory effect *in vitro*, including *Bacteroides distasonis*, *Bifidobacterium longum*, and *Blautia obeum* and led to decreased relative abundances of *F. prausnitzii*, *Clostridium cluster XIV*, and Enterobacteriaceae in human fecal cultures (Maier et al., 2018; Jia et al., 2019; O’Reilly et al., 2020). Several animal studies found differences in the microbiota composition after tacrolimus treatment, but findings on affected taxa differed between studies, possibly as a result of variations in dosage and administration (Supplementary Table 2; Bhat et al., 2017; Tourret et al., 2017; Jiang et al., 2018; Toral et al., 2018; Zhang et al., 2018; Han et al., 2019; Jiao et al., 2020). Two mice studies further investigated the impact of tacrolimus on microbial function. Jiao et al. (2020) found lower cecal concentrations of short chain fatty acids and Bhat et al. (2017) observed a lower abundance of microbial genes involved in pathways of sugar degradation, amino acid, fatty acid, lipid, and purine nucleotide biosynthesis and a higher abundance of genes involved in biosynthesis of cofactors, prosthetic groups, and electron carriers.

To our knowledge, no studies have been conducted investigating the effect of tacrolimus or cyclosporine A on the microbiota in IBD patients. However, in patients after heart transplantation, a higher diversity and an increased abundance of several bacterial taxa, such as Lachnospiraceae, *Blautia*, and *Roseburia*, were found in the higher dose (median dose 0.18 mg/kg/day) as compared to an increase in a taxon belonging to Bacteroides in the low dose (0.07 mg/kg/day) tacrolimus group (Jennings et al., 2020). However, many if not all patients used co-medication (Jennings et al., 2020), of which some can influence the intestinal microbiota, such as proton pump inhibitors (Imhann et al., 2017). Furthermore, the at least 4-fold higher plasma trough levels even in the low-dose group makes these data difficult to compare with the IBD patient group. Thus, before knowledge about the impact of calcineurin inhibitors becomes relevant for clinical implementation, research needs to focus on IBD patient populations, investigating relevant dosages. To this end, well-phenotyped IBD populations should be followed to analyze the fecal microbiota composition longitudinally before and during calcineurin inhibitor therapy.

## 5. Anti-TNF biologicals

TNF-α inhibitors, including infliximab, adalimumab, golimumab, and certolizumab-pegol are Immunoglobulin (IgG) 1 monoclonal or Fragment antigen binding (Fab) antibodies that are administered intravenously or subcutaneously (Levin et al., 2016). These agents bind to the soluble and transmembrane cytokine TNF-α, which plays an important role in the pathogenesis of IBD. It is assumed that clearance of the antibodies takes place *via* proteolytic catabolism following receptor-mediated endocytosis in the cells of the reticulum endothelial system (RES) (Ordás et al., 2012b). Subsequent inhibition of downstream TNF-α signaling induces T-cell apoptosis and induction of M2 wound-healing macrophages (Levin et al., 2016).

One well-known mechanisms for loss of response to anti-TNF agents is the development of anti-drug antibodies (Katsanos et al., 2019). These are formed against Fab 2, which causes an accelerated clearance *via* the RES. However, anti-drug antibodies cannot be detected in 10–60% of patients with loss of response to infliximab and low anti-TNF serum levels. This suggests other mechanisms to be involved as well (Ben-Horin and Chowers, 2011).

### 5.1. Impact of the intestinal microbiota on anti-TNF biologicals inactivation and treatment response

Several bacteria are able to neutralize human IgG by binding to their Fragment crystallizable (Fc) region (Frick et al., 1992; Labbé and Grenier, 1995; Leo and Goldman, 2009). In addition, some *Streptococci* were shown to degrade immunoglobulins *in vitro*, of which the IgG-degrading enzyme of commensal skin pathogen *Streptococcus pyogenes* (IdeS) is an extensively studied example (von Pawel-Rammingen et al., 2002; Wenig et al., 2004). IdeS-cleaved infliximab showed a decreased ability to bind complement factor C1q and Natural Killer (NK) cell receptor FCγRIIIA, which led to reduced FCγRIIIA-mediated NK-cell response *in vitro* (Supplementary Table 1; Deveuve et al., 2020). It can be expected that intestinal bacteria can exert comparable mechanisms and are able to degrade anti-TNF IgG antibodies or other IgG-based antibodies, such as the anti-α4β7 integrin vedolizumab (Wyant et al., 2016). Although parenterally administered anti-TNF agents may interact with the intestinal microbiota at the site of mucosal inflammation [i.e., intramucosal bacteria as reported for CD and UC (Moussata et al., 2011)] and can “leak” to the intestinal lumen (Derijks et al., 2018) likely facilitated by a disrupted barrier, it remains to be elucidated to what extent microbiota-driven degradation occurs in IBD patients and how relevant this is regarding treatment response.

Although data on the direct effects of microbes on anti-TNF agents in patients are lacking, several studies tried to predict anti-TNF treatment response based upon the pre-treatment microbiota composition. In a recent systematic review, the data of ten studies indicated that a higher baseline fecal or colonic abundance of Firmicutes, in particular Clostridiales, may be favorable for treatment response and sustained remission (Estevinho et al., 2020). A study on mucosal biopsies could identify further taxa that differed between anti-TNF responders and non-responders, including increased abundances of *Bifidobacterium*, *Collinsella*, *Lachnospira*, *Lachnospiraceae*, *Roseburia*, *Eggerthella* and reduced abundances of *Phascolarctobacterium* (Yilmaz et al., 2019). In a study on infliximab responders and non-responders, differences in the relative abundance of fecal bacterial taxa were also reported prior to the start of drug treatment, including higher levels of Enterobacteriaceae and Peptostreptococcaceae and lower levels of Clostridia in non-responders. The differences in fecal microbial profiles that distinguished infliximab responders and non-responders varied between UC and CD patients (Ventin-Holmberg et al., 2021), stressing the relevance of studying different disease phenotypes separately. Interestingly, all abovementioned studies could also relate lower abundances of short chain fatty acid-producing bacteria at baseline with treatment non-response (Supplementary Table 2; Yilmaz et al., 2019; Estevinho et al., 2020; Ventin-Holmberg et al., 2021).

Since the identification of microbial biomarkers that could predict treatment response in various studies is very valuable for IBD patient care, increasing the knowledge about causal relationships would make these associations more resilient. Therefore, future studies should focus on the ability of anti-TNF-microbiota interactions, applying for instance traceable biologicals to *in vivo* models and identifying potential binding to microbes. In addition, clinical relevance needs to be evaluated in different IBD populations.

### 5.2. Impact of anti-TNF biologicals on the intestinal microbiota

Since anti-TNF agents are highly specific to TNF-α, a direct effect on microbes seems unlikely. Still, a study in dextran sodium sulfate-treated mice showed increased abundances of *F. prausnitzii*, *Bacteroides*, Clostridiaceae, and Enterococcaceae (Petito et al., 2019). In IBD patients, some recent studies consistently showed an increase of the fecal microbial diversity and relative abundances of *Blautia* and *Roseburia* after infliximab therapy, which may be considered beneficial, while abundances of other taxa varied between the studies (Supplementary Table 2; Wang et al., 2018; Dovrolis et al., 2020; Kowalska-Duplaga et al., 2020; Zhuang et al., 2020; Ditto et al., 2021). However, it is not clear whether these changes result from a direct effect or an indirect effect, such as reduced intestinal inflammation, a restored intestinal barrier function, or altered dietary intake. Further insight in direct and indirect mechanisms between biologicals and the microbiota as well as clinical implementations of these findings remain to be investigated. *Ex vivo* fecal cultures can be used to elucidate direct interactions of anti-TNF agents with the IBD patient intestinal microbiota.

## 6. Tofacitinib

Tofacitinib is a highly selective inhibitor of mainly Janus kinase (JAK) 1 and JAK3. It can bind reversibly to the intracellular ATP binding site of JAK. The intracellular JAK1 and JAK3 signaling molecules are connected to transmembrane cytokine receptors on immune cells. Upon activation, JAK1-JAK3 pairing leads to STATs phosphorylation and can finally stimulate T-helper 17 cell proliferation and T-helper 1 cell activation, enhancing intestinal inflammation (Fernández-Clotet et al., 2018). Accordingly, inhibition of JAK1 and JAK3 by tofacitinib inhibits downstream phosphorylation of STATs, which leads to a reduction of cytokine production in immune cells and subsequent reduction of inflammation (Pfizer Limited, 2017; Fernández-Clotet et al., 2018). Additionally, tofacitinib seems to induce regulatory immune functions in human monocytes *ex vivo* (Cordes et al., 2020) and has been shown to repair cytokine-induced barrier dysfunction in epithelial cell culture models, targeting tight junction proteins (Sayoc-Becerra et al., 2020). Tofacitinib is a novel drug and so far recommended for the treatment of UC by the ECCO and American Gastroenterology Association guidelines (Harbord et al., 2017; Feuerstein et al., 2020).

Tofacitinib is administered orally and achieves 74% bioavailability. The majority is circulating as active compound, while about 35% is circulating as metabolites converted by the liver enzymes CYP3A4 and CYP2C19, which are predicted to have a 10-fold lower potency as compared to the parent drug (Dowty et al., 2014; Pfizer Limited, 2017). Several studies showed that the percentage of short- and long-term non-response is over 50% (Sandborn et al., 2012, 2017; Lair-Mehiri et al., 2020), but the potential underlying mechanisms are not clear.

### 6.1. Tofacitinib-microbiota interactions

Although tofacitinib is almost completely resorbed by the intestine, the remainder as well as different metabolites excreted *via* the liver may interact with the intestinal microbiota. However, to our knowledge, no studies are available on the conversion of tofacitinib by intestinal microbes and only one study has investigated the effect of tofacitinib on the intestinal microbiota in a murine experimental arthritis model (Hablot et al., 2020). This study showed a significant impact on the overall microbial community structure in mice treated with tofacitinib as compared to placebo (Supplementary Table 2; Hablot et al., 2020).

The drug targets, the JAK isoforms, are only conserved within the mammalian clade of Eutheria (EMBL-EBI, 2023; NCBI, 2023), which makes an interaction between tofacitinib and intestinal microbes yet only speculative. Nonetheless, due to its capacity to block the ATP binding site of JAK (Fernández-Clotet et al., 2018), tofacitinib may also interfere with microbial ATP binding sites. An *ex vivo* fecal culture study could be used to explore a potential impact on microbiota composition and function as well as potential microbial absorption, accumulation, and metabolism of tofacitinib. Following *in vitro* microbial culture studies can focus on underlying mechanisms of these potential interactions. In addition, IBD cohort studies may also screen for an impact of tofacitinib on microbiota composition and function and may link outcomes to clinical parameters, such as side effects.

## 7. Discussion

The aim of this review was to elucidate the evidence and clinical relevance of pharmacomicrobiomics in IBD. We summarized the current evidence on bi-directional drug-microbiota interactions for the most common IBD drugs and discussed their potential impact and implications for personalized medicine.

In general, the overall evidence varies between common IBD drugs. To date, there is some knowledge about the bacterial metabolism of thiopurines and MTX. Studies on 5-ASA and anti-TNF have mainly examined their impact on the microbiota composition or have aimed to predict treatment response based upon the intestinal microbiota composition.

The available evidence on drug-microbiota interactions generated so far stems from a variety of study designs and analysis methods. While this makes the direct comparison of study outcomes challenging, combined data from *in vivo*, *ex vivo*, as well as *in vitro* studies are essential to obtain further insight in consistency of findings, unraveling underlying mechanisms, and substantiating its clinical relevance.

### 7.1. Clinical relevance and personalized treatment

Combining the evidence, it is clear that the intestinal microbiota can influence the fate of some IBD drugs, such as MTX, or convert pro-drugs into the active compound, such as for thiopurines. Both may influence the systemic drug availability, while luminal drug activation also provides new opportunities for local treatment avoiding systemic side effects as has been proposed for thiopurines (Oancea et al., 2017). Herein, the individual microbiota composition will lead to individual differences in microbial drug metabolisms and subsequent gut availability. Even when causal relationships are largely unknown, the fecal microbiota may be valuable as non-invasive screening tool for the prediction of treatment response. However, microbial composition-based prediction models are so far not sufficiently reproducible. Therefore, specific microbial taxa or genes involved in drug metabolism should be identified to aid accurate prediction of treatment response and side effects, and by doing so, this may further optimize IBD treatment. In addition, complementary treatment based on the person-specific microbial needs may be included in personalized treatment, such as supplementation with specific microbial taxa that have anti-inflammatory properties or increase drug availability. Furthermore, genetically modified bacteria may be used for targeted drug delivery or pro-drug activation. Another complementary example is to administer the engineered probiotic *E. coli* Nissle 1917 that releases self-assembling therapeutic curli of trefoil factors, which aid in restoring the disrupted mucosal barrier (Praveschotinunt et al., 2019; Yu et al., 2020).

Besides, various drugs can impact microbiota composition and function. Potentially, these alterations could in turn influence host physiology, for instance by ameliorating intestinal inflammation or by altering hepatic metabolism (Björkholm et al., 2009; Ahmed et al., 2016). These effects can also be used to improve personalized medicine by administering (non-)IBD drugs that inhibit pro-inflammatory or stimulate anti-inflammatory or intestinal barrier enhancing microbial mechanisms.

### 7.2. Future directions

At the end of every chapter, we provided suggestions for future research to increase the understanding of drug-microbiota interactions of the particular drug, based on the current evidence. In general, future studies that investigate the impact of IBD drugs on the intestinal microbiota should focus on microbially comparable patient subgroups to draw firm conclusions, which includes the differentiation between UC and CD and other sub-phenotypes (Prosberg et al., 2016; Vich Vila et al., 2018; Galazzo et al., 2019). Furthermore, research increasingly points out that alterations in microbial function rather than only in microbiota composition are more relevant for interactions with the host in general and for inflammation in IBD specifically. Herein, innovative advanced techniques, which combine mechanistic and clinical parameters may additionally aid to benefit timely from new findings. For instance, integrated cross-kingdom transcriptomics and proteomics can detect changes in microbial function and host response during drug treatment, which can be directly related to patient outcomes (Westermann and Vogel, 2021). Another example is the so-called gut-on-a-chip *in vitro* microbiota co-culture model, which can be used to explore microbiota-drug-host interactions while modifying numerous parameters (Ashammakhi et al., 2020).

In conclusion, drug-microbiota interaction is a developing and promising field of study, which has potential to provide further insight in microbiota-related inter-individual differences in treatment response and side effects. Yet, evidence is not sufficient to directly implement knowledge on bidirectional drug-microbiota interactions into IBD patient care. More research is needed to find consent on microbial predictors for treatment response and side effects, to elucidate the clinical relevance and application of demonstrated *in vitro* or *ex vivo* drug-microbiota interactions in IBD. Ultimately, this may lead to identify targets for “complementary” or alternative intervention strategies to improve patient health outcomes.

## Author contributions

HB, DJ, and JP designed the study. HB and KD searched and evaluated relevant literature and wrote the manuscript. LD contributed with relevant pharmacological expertise. All authors revised the manuscript and approved the final version.
